# Benchmarking Multimodal Workload Classification: Effects of Modality, Validation Protocol, and Segmentation Contrast on an Open Graded-Arithmetic Dataset

**DOI:** 10.3390/bioengineering13070820

**Published:** 2026-07-16

**Authors:** Liam Booth, Adeel Mehmood, Mehdi Zeinali

**Affiliations:** 1 Faculty of Science and Engineering, University of Hull, Hull HU6 7RX, UK; m.zeinali@hull.ac.uk; 2 School of Digital and Physical Sciences, Faculty of Science and Engineering, University of Hull, Hull HU6 7RX, UK; a.mehmood@hull.ac.uk

**Keywords:** mental workload, EEG, ECG, pupillometry, multimodal fusion, machine learning benchmark, segmentation, cross-validation, open data, ds007262

## Abstract

Physiology-based mental workload classification is hard to compare across studies because task design, preprocessing, segmentation, and validation protocols vary widely. Using OpenNeuro ds007262, an open multimodal arithmetic dataset of synchronised 19-channel 10–20 system electroencephalography (EEG), electrocardiography (ECG), and pupillometry data from 18 released participants (16 retained after participant-level quality control for downstream modelling) spanning seven objective difficulty bands plus baseline fixation, we present a reproducible end-to-end machine learning pipeline for graded workload classification. The pipeline standardises participant-level quality control, trial-aligned windowing, modality-specific preprocessing and feature extraction (153 EEG, 18 ECG, and 25 pupillometry features), and supervised evaluation under three validation protocols (within-participant, pooled-stratified, and group-holdout) over eleven models and five class scenarios. Fused representations generally performed best; EEG was the strongest unimodal modality, and classical models outperformed deep models in most feature-based conditions. The best 6 s pipeline reached a balanced accuracy of 0.635; under denser 3 s overlap segmentation, the best pipeline reached 0.718. Mean balanced accuracy across 60 matched cells rose from 0.324 to 0.380, with gains concentrated in within-participant and pooled-stratified evaluation rather than strict unseen-participant transfer. The pipeline provides a transparent benchmark framework for fine-grained physiological workload modelling.

## 1. Introduction

Physiology-based mental workload classification remains difficult to compare across studies because task design, label construction, preprocessing, feature engineering, segmentation, and validation protocols vary widely across the literature. Recent reviews emphasise that the field still lacks standardised benchmark practices and that reported performance is often not directly comparable across datasets or evaluation schemes. This is particularly important in electroencephalography (EEG)-based workload modelling, where methodological variation can alter both absolute scores and model rankings, making reproducible benchmark design a central requirement rather than a reporting detail [1,2,3]. Recent primary studies reinforce this heterogeneity: workload classification has been reported with EEG connectivity features on open EEG datasets, multimodal EEG, and electrocardiography (ECG); pupillometry features in driving simulation; and integrated EEG–eye-tracking features in surgical training, each with different task structures, feature sets, and validation choices [4,5,6]. The comparison is therefore necessary because a single headline classifier score cannot show whether performance differences come from sensing modality, feature representation, temporal segmentation, validation protocol, or classifier family; evaluating these factors within one shared pipeline makes the source of the differences more interpretable. The present benchmark is grounded in an openly released multimodal arithmetic dataset with explicit event structure, difficulty labels, and Brain Imaging Data Structure (BIDS)-conformant organisation, where BIDS is a standard directory and metadata convention for organising neurophysiological recordings and event annotations. This allows methodological comparisons to be tied to a transparent shared data source [7].

The present study addresses this problem using OpenNeuro ds007262, an open multimodal arithmetic workload dataset containing synchronised EEG, ECG, and pupillometry recordings collected across seven objective arithmetic difficulty bands plus baseline fixation [7,8,9]. The dataset’s structured trial timing and graded task design make it well suited for controlled benchmarking of segmentation, modality choice, fusion strategy, and validation regime under a shared pipeline. By using an openly available dataset with explicit benchmark factors, the present work aims to support more transparent comparison than is typical in workload-classification studies built around single closed datasets or isolated modelling claims [2,3,7].

This paper therefore treats two design choices as benchmark variables rather than secondary implementation details: validation regime and temporal segmentation. Validation regime determines whether a score reflects personalised decoding, mixed-participant discrimination, or transfer to unseen individuals. Temporal segmentation determines the effective unit of analysis and the degree of temporal dependence between samples. Both choices are known to materially affect reported workload-classification performance, and both are evaluated here under otherwise matched preprocessing, feature extraction, and model selection settings [1,10,11].

The contribution of this paper is a reproducible benchmark for graded physiological workload classification on ds007262. We compare unimodal EEG, ECG, and pupillometry features against early-fusion representations within a shared end-to-end pipeline, and we evaluate how conclusions change across three validation targets: within-participant decoding, pooled mixed-participant discrimination, and stricter participant-aware testing. We further compare a single 6 s trial window against denser overlapping 3 s windows to test whether temporal resampling changes benchmark conclusions when the underlying trial structure is held constant. This framing positions the paper as a methodological benchmark study rather than a single-model performance claim [3,11].

## 2. Materials and Methods

Two methodological issues are especially relevant to this benchmark. First, mental workload studies remain difficult to compare because task type, class definition, and evaluation criteria vary substantially [1,2,3]. Second, segmented physiological data are vulnerable to overly optimistic evaluation when dependent observations from the same participant appear in both training and test sets. These concerns are directly relevant here because the present benchmark compares both pooled mixed-participant and participant-grouped evaluation regimes and contrasts single full-trial windows with overlapping sub-windows derived from the same parent interval.

### 2.1. Data Source and Pipeline Overview

Original recordings are available on OpenNeuro (accession ds007262) [7] as a Brain Imaging Data Structure (BIDS)-conformant [12] dataset containing EEG, ECG, and pupillometry streams synchronised through Lab Streaming Layer (LSL) and the acquisition protocol used to time-align multimodal recording streams [13], together with complementary marker streams used for event annotation. Before BIDS conversion, the synchronised raw streams were stored in Extensible Data Format (XDF), the file format used by the LSL LabRecorder application to save synchronised raw data streams and marker streams in a single recording file. The classification pipeline consumes these derivatives to build trial tables, apply quality control (QC), segment analysis windows, extract features, and construct machine learning datasets for benchmarking.

The underlying arithmetic paradigm was selected because it permits objective difficulty control rather than relying solely on subjective task labels. This control is implemented through *Q*-value stratification, where *Q*-value is an arithmetic item-difficulty descriptor derived from the structure of the addition problem, including interacting digits, digit size, and carry operations. Questions were generated across seven *Q*-value bands and presented in randomised order; randomising stimulus order across difficulty bands, rather than blocking by difficulty, dissociates nominal difficulty from linear time-on-task and habituation effects [7,8,9]. This makes the dataset particularly suitable for benchmarking graded workload estimation rather than only baseline-versus-task discrimination. This use follows Thomas’s communication theory account of calculation complexity [14]. The seven nominal bands used here were $Q_{1}=0.6$–1.5, $Q_{2}=1.5$–2.4, $Q_{3}=2.4$–3.3, $Q_{4}=3.3$–4.2, $Q_{5}=4.2$–5.1, $Q_{6}=5.1$–6.0, and $Q_{7}=6.0$–6.9, ordered from easiest to hardest.

**Figure 1:**
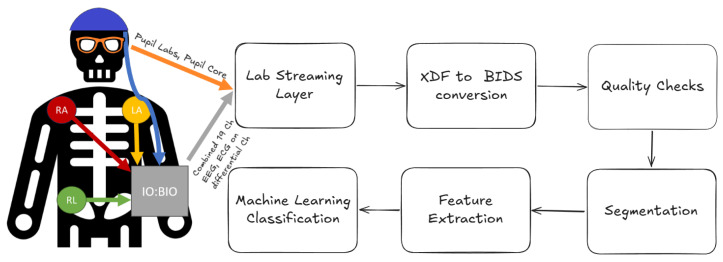
Data capture to classification pipeline: top-level diagram.

Figure 1 summarises the end-to-end flow from multimodal acquisition to workload classification. After BIDS event parsing, the pipeline screens participants and trials using modality-specific quality metrics, segments trial-aligned analysis windows, extracts unimodal features, constructs unimodal and early-fusion feature matrices, and evaluates classifiers under multiple validation protocols.

The pipeline is designed as a benchmarking framework rather than a single fixed classifier. Each stage produces explicit intermediate artefacts, retention summaries, and reproducible modelling tables so that downstream performance can be interpreted in the context of data inclusion, preprocessing choices, feature construction, and evaluation design.

The original LSL acquisition step (raw Extensible Data Format (XDF) capture and BIDS conversion) is documented separately as part of the dataset release [7] and is not re-executed by the analysis pipeline. The benchmark pipeline begins at Stage 0 (canonical trial-table construction from the BIDS derivatives). Table 1 summarises each pipeline stage and its key outputs.

The following sections describe how participant-level screening and window-level rejection rules determine which data contribute to each modelling condition. Because rejection is modality-specific, participant inclusion can differ across unimodal and fused benchmarks.

### 2.2. Quality Checks

Initial quality checks are performed at the participant level. Dropped-sample events and basic signal summaries are aggregated across trials to identify recordings with unreliable EEG or ECG acquisition, while pupil-detection confidence is summarised to flag low-quality eye-tracking sessions. These metrics define modality-specific carry-forward participant sets used in downstream windowing and modelling. Participant-level screening is applied before feature extraction so that benchmark differences are not dominated by obviously failed recordings. Because signal failure is modality-specific, a participant may be retained for one unimodal benchmark while being excluded from another or from the fused dataset. Thresholds were selected as conservative, reproducibility-oriented screening criteria rather than optimised hyperparameters [1,2].

Twenty participants were originally enrolled for ds007262. Two (sub-002 and sub-017) were excluded prior to release of the present benchmark dataset because of incomplete recordings or an incomplete arithmetic block, leaving 18 participants entering the analysis pipeline. Table 2 summarises the per-participant acquisition statistics for the EEG/ECG mobile recording device. Of these 18, the Stage 1 QC gate subsequently rejected sub-006 (anomalous event-marker count; included in Table 2 for audit) and sub-008 (excessive dropped-sample rate plus low pupil-detection confidence), leaving 16 participants carried forward for any unimodal or fused downstream modelling.

Thus, the participant counts refer to three distinct stages: 20 participants were originally recorded, 18 released participants entered the benchmark pipeline, and 16 participants passed the Stage 1 union carry-forward QC gate before modality-specific downstream exclusions were applied.

**Figure 2:**
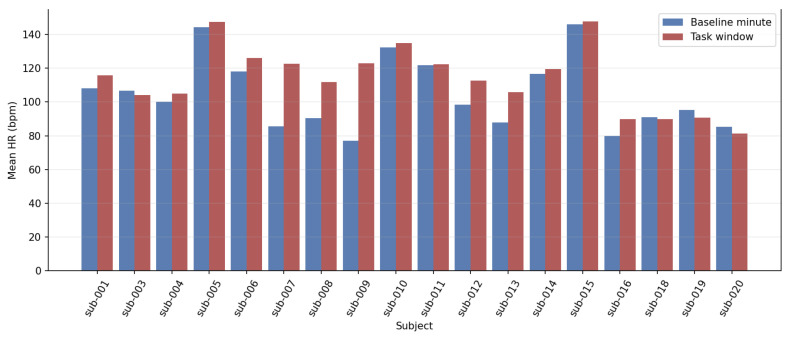
Baseline vs. task: mean heart rate by participant (n=18 released participants; bars show the 60 s fixation baseline and the averaged arithmetic task window).

As a simple physiological sanity check in the finished 6 s baseline run, mean heart rate was lower during the inter-trial fixation baseline than during task performance (104.7 bpm versus 113.3 bpm across participants); the elevated absolute baseline value was consistent with the baseline collected immediately adjacent to active arithmetic trials rather than that as a separate resting block. The within-participant baseline–task contrast was statistically significant across the 13 retained ECG participants (paired Wilcoxon signed-rank W=11, two-sided p=0.013; paired *t*-test p=0.045), with the mean rate higher during the task in 10 of the 13 participants. The participant-level baseline–task contrast is shown in Figure 2.

All participants completed the same seated, screen-based arithmetic protocol, so posture and gross physical activity were held constant across the sample. Two participants (sub-005 and sub-015, the two youngest, aged 21–22 years) show markedly higher mean rates (146 and 148 bpm). These are genuine elevations rather than a heart-rate-doubling detection artefact: for the cleanly measured sub-015 only 1.6% of instantaneous values fall outside the physiological 35–180 bpm range, its 5th–95th-percentile range is 111–176 bpm, and an estimate from detected beats divided by recording duration (144 bpm) matches the windowed mean (148 bpm); sub-005 gives the same robust central picture (median 154 bpm, beats-per-duration 150 bpm) but with a noisier trace (29% of values out of range), so its mean is treated as less precise. We interpret both as genuine between-participant variation in tonic cardiac arousal under a demanding task. Conversely, three of the 13 retained participants show a slightly lower task than baseline mean; sub-019 is one such case (−4.6 bpm), a difference that is small relative to that participant’s own beat-to-beat variability (5th–95th-percentile instantaneous rate 71–155 bpm) and so reflects noise around no change rather than a meaningful reversal. The neighbouring sub-020 in Figure 2 (−3.9 bpm) was retained in the union Stage 1 gate but excluded from the ECG modelling set by the modality-specific dropped-sample screening (its 95th-percentile per-trial dropped-sample count exceeded the threshold), so it is shown for completeness but does not enter the ECG test. Because the baseline is an inter-trial fixation minute recorded immediately adjacent to active trials rather than a separate resting block, it is already mildly elevated, which makes such small individual reversals unsurprising.

**Figure 3:**
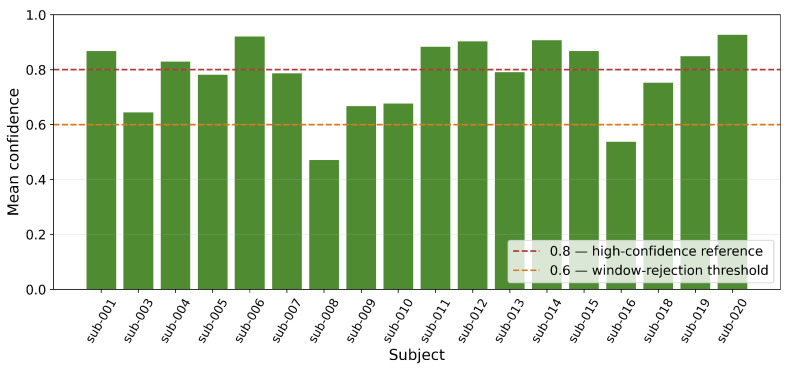
Pupillometry average confidence by participant. Dashed lines mark the operative window-rejection threshold (0.6) and a high-confidence reference level (0.8); only the 0.6 level is used to reject data.

Pupil Labs confidence values range from 0 to 1 and provide a per-sample estimate of pupil-detection reliability. The pipeline first summarises confidence at participant level and then applies window-level rejection when mean confidence falls below 0.6, effective coverage is too low, or the proportion of low-confidence samples exceeds a configured threshold (Figure 3). In Figure 3 the dashed line at 0.6 marks this operative window-rejection threshold, below which a window is discarded. The dashed line at 0.8 is a descriptive high-confidence reference only (the level above which Pupil Labs regard detection as high quality); the pipeline additionally reports the percentage of samples below 0.8 as a stricter quality descriptor, but 0.8 is not itself used as a rejection criterion.

### 2.3. Preprocessing

EEG was recorded from 19 scalp electrodes arranged according to the international 10–20 system at 250 Hz with a linked-ear reference (electrodes Fp1, Fp2, F7, F3, Fz, F4, F8, T3, C3, Cz, C4, T4, T5, P3, Pz, P4, T6, O1, and O2; Ag/AgCl with conductive gel), recorded on a combined LSL-integrated EEG/ECG mobile system [16,17]; ECG was recorded as a synchronised three-lead montage on the same device, sampled at 250 Hz (the same device and rate as the EEG). The montage comprises right-arm (RA) and left-arm (LA) sensing electrodes together with a right-leg (RL) electrode, which serves as the driven ground/reference that suppresses common-mode interference rather than as a measurement lead; the RA, LA, and RL positions are those shown on the body in Figure 1. Pupil diameter and gaze were recorded using a Pupil Labs Pupil Core eye tracker (Pupil Labs, Berlin, Germany) [18]. All modalities were synchronised through the Lab Streaming Layer protocol, which streamed each device over a UDP-based transport on the local Wi-Fi network; occasional Wi-Fi packet loss therefore caused brief gaps in the EEG/ECG stream, but the mobile device’s monotonic per-sample counter allowed the XDF-to-BIDS converter to detect, count, and flag the missing samples deterministically, rather than it silently interpolating them. These flagged samples are the “dropped” counts reported in Table 2 and were excluded from epoching. Preprocessing was performed in MNE-Python (version 1.11.0) [19]. A 50 Hz notch filter removed powerline interference, followed by a 3rd-order Butterworth IIR bandpass filter between 2 and 40 Hz. Bad channels were identified using a median absolute deviation (MAD)-based amplitude criterion (channels exceeding 5× the median standard deviation) and interpolated using spherical splines. Signals were then re-referenced to the common average. Independent component analysis (ICA) was not applied in the reported runs; this choice prioritises pipeline simplicity and reproducibility over per-recording artefact removal, and the impact of optionally enabling ICA is left as an explicit sensitivity analysis for future work.

ECG signals were median-centred and filtered using two 2nd-order Butterworth bandpass filters applied via zero-phase filtfilt: a broad band (0.5–40 Hz) for cleaned waveform output and a narrower peak-detection band (5–25 Hz) for R-peak identification. R-peaks were detected using SciPy’s find_peaks with a MAD-based adaptive prominence threshold and a minimum inter-peak distance of 0.3 s; instantaneous heart rate values outside 35–180 bpm were excluded from feature computation.

Pupillometry samples with Pupil Labs confidence below 0.6 or a non-positive diameter were marked invalid and linearly interpolated. A moving-average filter with a 0.2 s window was applied for smoothing, and traces were resampled to 100 Hz via linear interpolation.

### 2.4. Segmentation

Two windowing configurations are evaluated. The baseline profile uses one fixed 6 s calculation window aligned to the arithmetic presentation period, matching the trial structure of ds007262. The overlap profile partitions the same 6 s parent interval into three 3 s sub-windows with a 1.5 s step, increasing temporal sampling density while preserving the same underlying trials. Sliding-window approaches of this kind are common in EEG-based classification because they can improve temporal localisation of discriminative signal components and support more frequent decision updates. However, overlapping sub-windows derived from the same parent trial are not statistically independent observations and should therefore be interpreted as denser temporal sampling rather than as a proportional increase in independent evidence [20].

### 2.5. Feature Extraction

The three modalities were retained because they offered partly complementary access to workload-related physiology. EEG provides the most direct measure of task-related cortical dynamics, ECG-derived measures capture autonomic adjustment associated with cognitive effort, and pupillometry provides a sensitive ocular index long associated with mental processing demand [9,21,22,23,24]. Benchmarking each modality independently as well as in fusion therefore allows the present study to distinguish whether improved performance reflects genuinely complementary information or simply the dominance of one signal family.

Features are computed for each retained window or sub-window rather than from participant-level trial summaries. Separate EEG, ECG, and pupil feature tables are produced first, after which unimodal datasets (EEG-only, ECG-only, pupil-only) and early-fusion datasets are assembled by joining windows that share the same trial and window key.

**Figure 4:**
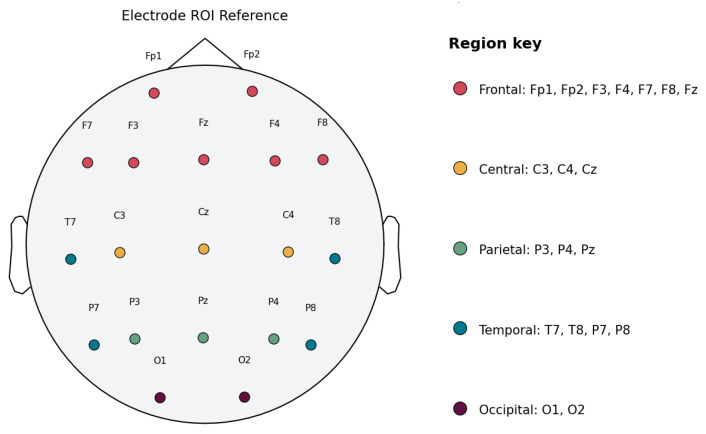
EEG electrode region-of-interest reference.

EEG contributes 153 engineered features, dominated by spectral band power summaries, including absolute and relative power measures and band ratios across five canonical bands (delta 1–4 Hz, theta 4–8 Hz, alpha 8–13 Hz, beta 13–30 Hz, high-beta 30–40 Hz), together with time-domain and Hjorth descriptors. The reported count is obtained from five fused compatible ROIs, each with 27 ROI-level EEG summaries (5×27=135), plus 15 ROI-by-band baseline-delta features and 3 frontal/Fz channel-specific features, giving 153 EEG input features; the full per-modality count derivation is given in Table A1. Most EEG features are aggregated across five regions of interest (ROIs)—frontal (Fp1, Fp2, F3, F4, F7, F8, Fz), central (C3, C4, Cz), parietal (P3, P4, Pz), temporal (T3/T4 and T5/T6 normalised to the 10–10 labels T7/T8 and P7/P8), and occipital (O1, O2)—while three features remain channel-specific: frontal alpha asymmetry (F4 and F3) and absolute and relative theta power at Fz. The ROI grouping used in the current implementation is shown in Figure 4. Frontal alpha asymmetry is computed as $\ln\left({\mathrm{alpha}}_{F4}\right)-\ln\left({\mathrm{alpha}}_{F3}\right)$; under this sign convention, positive values indicate relatively greater right-frontal alpha and are typically interpreted as relatively reduced right-frontal cortical activation. The full list of EEG features is provided in Table A2 in Appendix A.

ECG contributes 18 features capturing heart rate (HR) and heart rate variability (HRV) summaries, including RR-interval statistics, the root mean square of successive differences (RMSSD), the standard deviation of NN intervals (SDNN), NN50/pNN50, and basic quality and validity indicators; the full set is given in Table A3 in Appendix A.

Pupillometry contributes 25 features summarising pupil size dynamics, gaze behaviour, and signal validity, including distribution summaries, baseline-referenced change measures, velocity-derived metrics, confidence estimates, and valid-coverage indicators; the full set is given in Table A4 in Appendix A.

The feature counts reported in the abstract and results are therefore feature-column counts after excluding non-feature metadata: 153 EEG features, 18 ECG features, and 25 pupillometry features; the early-fusion models use the concatenation of these columns, giving 196 fused input features. Appendix A gives both the feature equations and a count summary so that the reported dimensionalities can be reproduced from the implemented feature groups. For code-level reproducibility, the archived pipeline release implements modality-specific feature extraction in stage4_extract_features.py and fused-table assembly in stage5_build_fused_table.py [15].

### 2.6. Machine Learning

The benchmark evaluates three distinct generalisation targets rather than minor cross-validation variants. Within-participant evaluation trains and tests within one participant at a time using an internal stratified split and therefore estimates personalised decoding performance after participant-specific calibration. Pooled-stratified evaluation pools rows across participants and applies stratified row-level splits, preserving class balance while allowing the same participant to contribute rows to both training and test partitions. Group-holdout evaluation keeps participants separated between training and test sets and therefore provides the strictest estimate of transfer to unseen participants.

These regimes should be interpreted separately. Within-participant results quantify personalised decoding under participant-specific calibration. Group-holdout results quantify transfer to unseen participants and are the only protocol whose scores can be cited as evidence of subject-independent generalisation. Pooled-stratified results are explicitly included here as a controlled illustration of how ignoring the participant grouping when splitting samples inflates apparent performance, because dependent observations from the same individual appear in both training and test partitions; we therefore caution against citing pooled-stratified scores as evidence for deployment-ready workload classifiers, and report them mainly as a contrast to the participant-aware protocols [10,25].

Eleven candidate models were evaluated using scikit-learn (version 1.8.0) [26] for the classical pipelines and PyTorch (version 2.6.0) [27] for the four neural models. The candidate set was chosen to cover representative baseline model types commonly used for tabular physiological workload features: a linear baseline (logistic regression), a kernel method (RBF-SVM), a probabilistic baseline, tree-based and ensemble methods, distance-based learning, shallow neural learning, and recurrent, convolutional, and attention-based neural classifiers, all evaluated under the same feature tables and validation protocols, consistent with recent workload-classification studies that compare classical tree, kernel, and ensemble methods against neural or multimodal alternatives [4,5,6]. The grids listed below summarise the principal hyperparameter axes; the complete search-space definition is reproduced verbatim in analysis_pipeline/stage6_train_classic_ml.py.

1. Logistic regression (LBFGS solver, C∈{0.1,1.0,5.0,10.0}, balanced class weights);
2. Support vector machine (SVM; RBF kernel, C∈{0.5,1.0,3.0,10.0}, γ∈{scale,auto});
3. Gaussian naïve Bayes (var_smoothing $\in \{10^{-9},10^{-8},10^{-7}\}$);
4. Decision tree (Gini/entropy, max depth ∈{None,8,16,24}, min_samples_leaf∈{1,2,4}, balanced class weights);
5. *k*-nearest neighbours (KNNs; k∈{3,5,9,13}, weights∈{uniform,distance}, p∈{1,2});
6. Multilayer perceptron (MLP; hidden layers ∈{(64,),(128,),(64,32)}, $\alpha \in \{10^{-4},10^{-3}\}$, learning_rate_init$\in \{10^{-3},5\times 10^{-4}\}$);
7. Random forest (RF; 400 trees, balanced-subsample weighting;
  max depth ∈{None,10,20}; min_samples_leaf∈{1,2,4};
  max_features∈{sqrt,0.5});
8. Long short-term memory (LSTM; PyTorch, hidden size ∈{64,96}, layers∈{1,2});
9. Gated recurrent unit (GRU; PyTorch, hidden size ∈{64,96}, layers∈{1,2});
10. One-dimensional convolutional neural network (1D-CNN; PyTorch);
11. Transformer (PyTorch, $d_{\mathrm{model}}\in \left\{32,64\right\}$, layers∈{1,2}).

All neural models consume the engineered feature vector (153 EEG, 18 ECG, 25 pupillometry, or 196 fused features) treated as a 1D sequence with a sequence length equal to the feature count and an input channel size of 1. The CNN1D applies three Conv1D blocks (channels 1→16→32→32, kernel sizes 5/5/3, ReLU + BatchNorm), adaptive average pooling to length 16, and a two-layer MLP head (Linear → ReLU → Dropout → Linear; default hidden dimension 64). The LSTM and GRU each instantiate nn.LSTM/nn.GRU with input_size=1, take the final time-step embedding, and decode through a two-layer MLP head with the same activation pattern.

**Figure 5:**
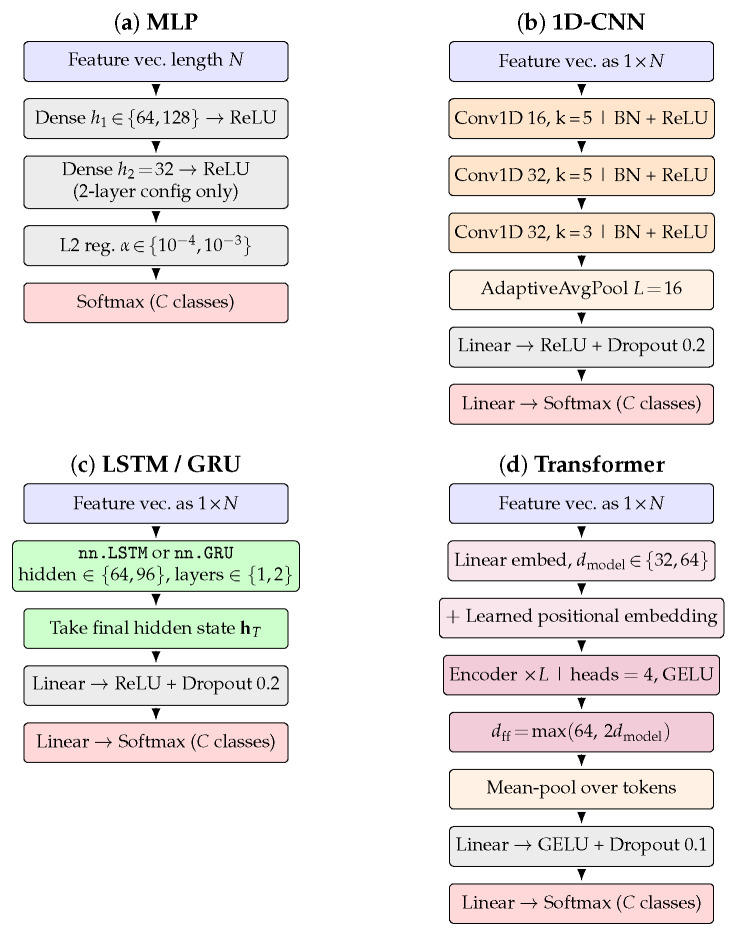
Block-diagram structure of the four neural architectures evaluated, ordered from simplest to most complex: (**a**) MLP, (**b**) 1D-CNN, (**c**) LSTM and GRU (nn.LSTM/nn.GRU are interchangeable and share an identical hidden-size and layer-count search grid plus the same two-layer MLP head), and (**d**) transformer. Inputs in all cases are the engineered feature vector (EEG, ECG, pupillometry, or fused) of length N∈{18,25,153,196} treated as a sequence of unit-channel tokens for the convolutional, recurrent, and attention models. Hyperparameter ranges shown indicate the discrete search grids; per-fold selection used inner 3-fold stratified cross-validation over up to six randomly sampled configurations. The MLP shown is the sklearn feature-vector classifier; deep models are PyTorch  implementations.

The transformer projects each feature value to a $d_{\mathrm{model}}=64$ token via a learned linear embedding, adds a learned positional embedding of length equal to the feature count, applies a TransformerEncoderLayer stack ($n_{\mathrm{heads}}=4$, GELU activation, $d_{\mathrm{ff}}=\max\left(64,2d_{\mathrm{model}}\right)$), mean-pools across tokens, and decodes through a two-layer MLP head with GELU activation. All neural models were trained with AdamW (learning rate $10^{-3}$, weight decay $10^{-4}$), CrossEntropyLoss with class-balanced weighting, 30 training epochs, batch size 64, dropout 0.2 (0.1 for the transformer), and gradient clipping at unit norm. Hyperparameters were selected via inner 3-fold stratified cross-validation over up to 6 randomly sampled parameter combinations per model (YAML-configured inner_folds: 3 and max_param_combos: 6). Features were preprocessed within each fold using a pipeline of median imputation, quantile clipping at the 1st and 99th percentiles, robust scaling (median/interquartile range (IQR) centring), and zero-variance filtering; no additional feature selection was applied. For within-participant evaluation, a single stratified 80/20 train–test split was used within each eligible participant. Pooled-stratified evaluation used 5-fold stratified cross-validation with a 20% held-out test fraction. Group-holdout evaluation used a leave-2-participants-out scheme repeated 5 times, yielding 15 group-holdout outer folds. All splits used a fixed random seed of 42 for reproducibility. Figure 5 summarises the architectures of the four neural models evaluated.

## 3. Results

### 3.1. Class-Construction Scenarios

**Figure A1:**
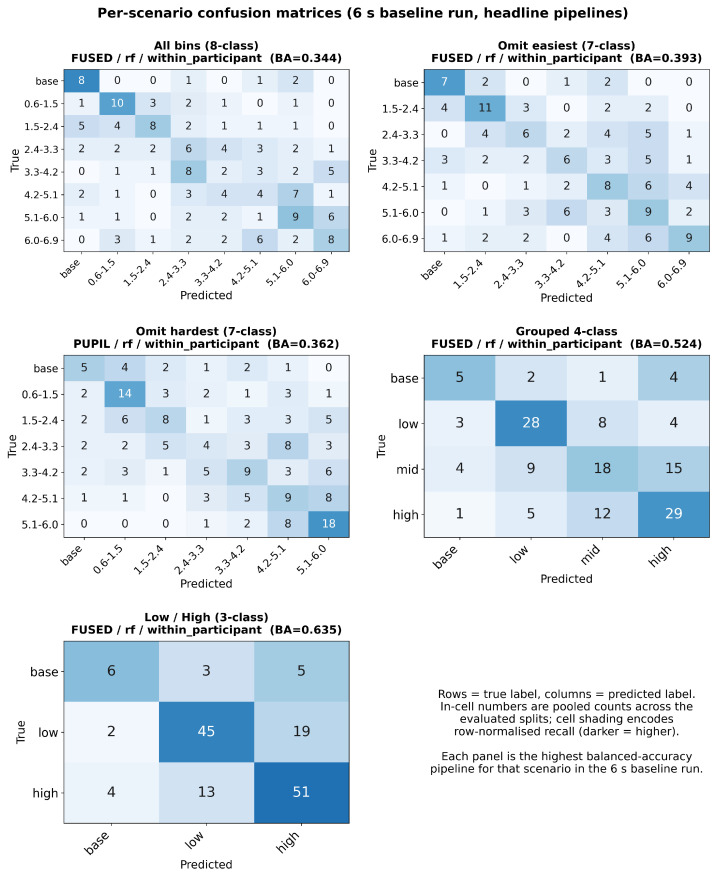
Confusion matrices for the highest balanced-accuracy pipeline of each of the five class-construction scenarios (6 s baseline run). Rows are true labels and columns are predicted labels; in-cell numbers are pooled counts and shading encodes row-normalised recall. Panel titles give the winning dataset, model, validation protocol and balanced accuracy (BA).

Five label-space constructions are evaluated in parallel. They are designed to test the sensitivity of benchmark conclusions to class cardinality and to the inclusion of the most extreme difficulty bands, rather than to claim a single “correct” workload labelling. Table 3 summarises the mapping from the seven *Q*-value difficulty bands plus baseline fixation onto the class labels used in each scenario. The reduced low_high scenario serves as the clearest performance endpoint, while the higher-cardinality scenarios provide more stringent tests of fine-grained workload discrimination. This table is retained because it defines the label space for every downstream benchmark comparison; confusion matrices answer a different, model-specific question about classification errors and are therefore shown for the headline pipelines rather than used as a replacement for the scenario-definition table. Confusion matrices for the headline (highest balanced-accuracy) pipeline of each of these five scenarios are additionally provided in Appendix C (Figure A1), so that the per-scenario error structure can be inspected directly.

### 3.2. Current 6 s Baseline Run Findings

Each class-scenario run evaluated the same analysis grid: four datasets (EEG, ECG, pupil, fused), three validation protocols, one feature-selector setting, and eleven candidate models. This produced 132 aggregate dataset–protocol–model comparisons and 1463 evaluated train/test executions per scenario in the completed 6 s baseline run.

#### 3.2.1. Data Retention and Filtering

Applying the Stage 1 union participant-level quality-control gate retained 16 of the 18 released participants (sub-006 and sub-008 were rejected outright). From these 16, modality-specific screening then defined the per-modality sets: EEG and ECG each retained 13 participants (excluding sub-011, sub-013 and sub-020), pupillometry retained 15 (excluding sub-016), and the fused set retained 12 (the intersection of the three, excluding sub-011, sub-013, sub-016 and sub-020).

Windowing yielded 1120 fixed 6 s epochs, of which 1051 were retained as multimodal rows after window-level rejection. Window-level rejection affected 10 EEG windows, 10 ECG windows, and 59 pupil windows, making pupil quality the dominant source of window loss.

**Figure 6:**
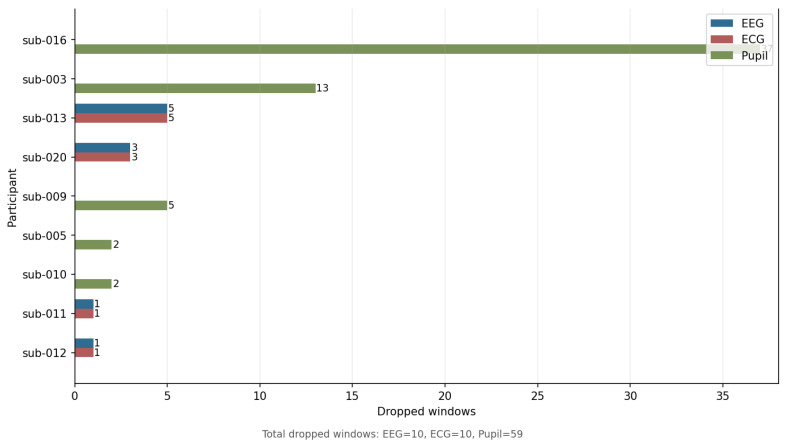
Per-participant breakdown of dropped windows due to missing samples during EEG/ECG segmentation.

The largest dropped-window burdens occurred for sub-016 (37 dropped windows, all pupil), sub-003 (13, all pupil), and sub-013 (10, split across EEG and ECG); Figure 6 shows the per-participant breakdown of these dropped windows.

Feature extraction produced 1110 EEG rows, 1110 ECG rows, and 1061 pupil rows across the 16 carried-forward participants. EEG and ECG remained within a relatively tight 65–70 row range per participant, whereas pupil ranged from 33 to 70 because of window-level confidence and coverage rejection, most notably for sub-016.

After modality-specific subject filtering and fused-table intersection, the machine learning tables contained 901 EEG rows, 901 ECG rows, 997 pupil rows, and 811 fused rows. The fused dataset omitted four participants (sub-011, sub-013, sub-016, sub-020), compared with three being omitted from EEG (sub-011, sub-013, sub-020), three from ECG (sub-011, sub-013, sub-020), and one from pupil (sub-016).

The current machine learning tables expose 153 EEG features, 18 ECG features, 25 pupil features, and 196 fused features after excluding non-feature metadata columns.

#### 3.2.2. Scenario Comparisons

The class scenarios are included to test how sensitive benchmark conclusions are to label-space construction rather than to present five equally weighted headline outcomes. The main question is whether the same broad modelling preferences remain visible as the discrimination problem becomes easier through class reduction or regrouping.

Across class scenarios, performance improved as the label space was simplified, confirming that finer-grained workload discrimination remains substantially harder than reduced-class formulations. Mean best balanced accuracy (BA) rose from 0.230 in the full all-bins setting to 0.505 in the reduced low-versus-high scenario, but the all-bins condition remained tractable rather than degenerate, with its best result reaching 0.344 under fused within-participant modelling.

Scenario-specific winners broadly favoured fused data and within-participant evaluation. Four of the five scenario winners used fused representations, and all five were obtained under within-participant evaluation. The main exception to the fused pattern was baseline omit hardest, where pupil alone produced the top score.

The strongest result in the completed 6 s baseline run was fused random forest under within-participant evaluation in the low-versus-high scenario omitting the hardest band, reaching balanced accuracy of 0.635 and macro-F1 of 0.601.

#### 3.2.3. Modality and Protocol Comparisons

Two benchmark-level questions are especially informative here: whether multimodal fusion outperforms unimodal inputs, and whether more permissive evaluation settings yield higher apparent performance than stricter participant-aware splits. The results support both expectations, with fused representations performing best overall and within-participant decoding producing the highest average scores. Multimodal physiological monitoring is widely used because different sensing streams capture partially complementary aspects of workload, while recent benchmark and reproducibility papers argue that evaluation design must be made explicit if scores are to be interpretable across studies [2,3,22,23].

At the modality level, fused data achieved the highest average best-balanced accuracy, followed by EEG, pupil, and ECG. The same broad ordering appeared in the best observed scores, indicating that multimodal fusion was generally most informative, EEG was the strongest unimodal signal, pupil remained useful, and ECG was consistently the weakest standalone modality in the present benchmark.

Fused data dominated most of the search space, accounting for 15 of the 60 dataset–protocol–scenario winner cells and four of the five overall scenario winners. EEG was the strongest unimodal modality and was also the best-performing modality under pooled evaluation, while pupil was the only unimodal signal to outperform fused in any scenario winner cell, specifically baseline omit hardest. ECG did not win any scenario and remained the weakest modality throughout.

Protocol-wise, within-participant evaluation gave the highest average best-balanced accuracy at 0.382, followed by pooled-stratified evaluation at 0.321 and group-holdout evaluation at 0.269. This pattern is consistent with the broader literature showing that less restrictive splits often produce more optimistic estimates than participant-aware testing, particularly when repeated or temporally related samples can appear across training and test partitions [10,11,25].

For pooled evaluation specifically, EEG was the strongest modality in the completed 6 s run. The top pooled-stratified result was EEG with support vector machine in baseline low high omit hardest at 0.588, whereas the strongest group-holdout result was fused with support vector machine in the same scenario at 0.500. By contrast, fused data remained strongest in the within-participant setting.

The highest-performing pipelines were dominated by classical models rather than deep networks. In this benchmark, random forest, support vector machine, logistic regression, and decision tree variants remained more reliable than the tested neural architectures across most feature-based conditions. This is compatible with benchmark work showing that simpler models can remain competitive, or even preferable, when sample sizes are modest, features are heavily engineered, and the main source of variance is methodological rather than architectural [3,28].

Across all dataset–protocol–scenario comparisons, classical models won 47 of 60 best-model cells, while neural models won 13. Classical models also achieved a higher mean family-best balanced accuracy (0.322 versus 0.295). Neural wins were concentrated in narrower parts of the search space, especially ECG and pooled-stratified cells. By dataset, fused was won 14 times by classical models and once by a neural model; EEG 12 versus 3; pupil 15 versus 0; and ECG 6 versus 9. By protocol, within-participant was 18 classical versus 2 neural, pooled-stratified 12 versus 8, and group-holdout 17 versus 3. Across all 60 dataset–protocol–scenario best-model cells, random forest accounted for 30 wins, compared with 9 for support vector machine, 7 for multilayer perceptron, 4 for transformer, and 4 for logistic regression. KNN appeared twice, while GRU, CNN, decision tree, and Gaussian naïve Bayes each appeared once. The strongest classical result was fused with random forest under within-participant evaluation in baseline low high omit hardest at a balanced accuracy of 0.635.

#### 3.2.4. Benchmark Performance Summary

The full per-cell summary of best-performing pipelines for each scenario–dataset–protocol combination in the 6 s baseline run is given in Table A5 (Appendix B) to keep the main text focused on aggregate-level patterns.

Table 4 summarises the mean best-balanced accuracy by modality and protocol, averaged across all five class scenarios in the 6 s baseline run. To address the request for variability measures, each modality–protocol cell also reports the sample standard deviation (SD), standard error (SE), and 95% confidence interval across the five class-scenario means.

Table 5 compares classical machine learning models against neural architectures across all 60 dataset–protocol–scenario best-model cells.

**Figure 7:**
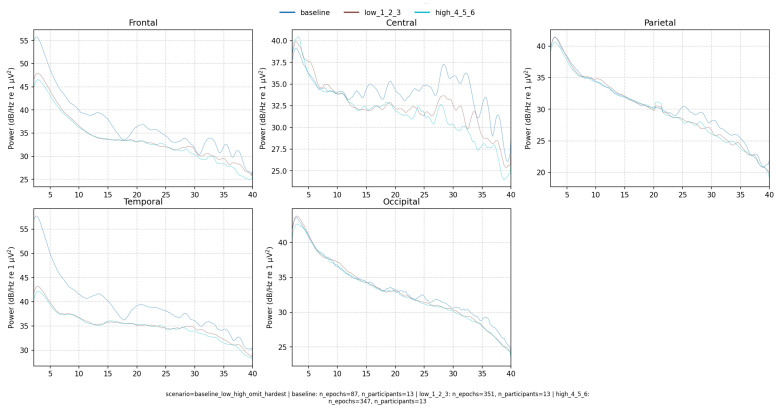
EEG power spectral density (PSD) by condition (baseline, low, high). Low corresponds to $Q_{1}$–$Q_{3}$ and high corresponds to $Q_{4}$–$Q_{6}$ in the reduced low-versus-high scenario, with $Q_{7}$ omitted.

**Figure 8:**
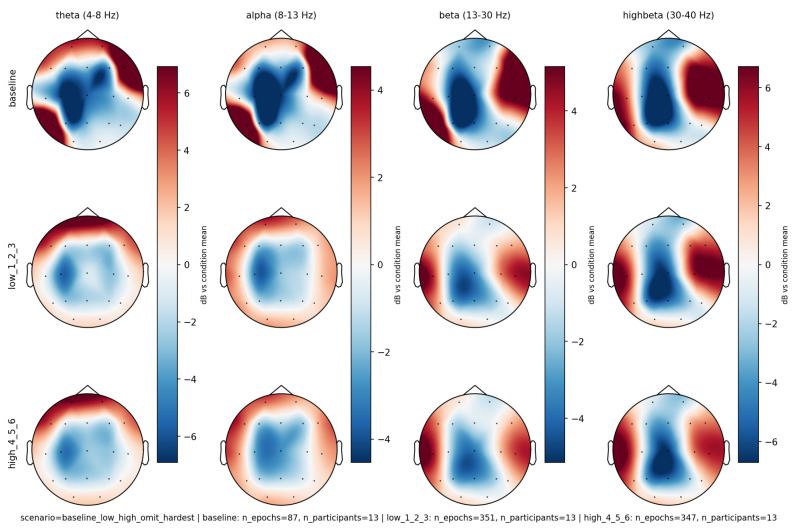
EEG topographic power maps by condition (baseline, low, high). Low corresponds to $Q_{1}$–$Q_{3}$ and high corresponds to $Q_{4}$–$Q_{6}$ in the reduced low-versus-high scenario, with $Q_{7}$ omitted.

To relate these benchmark outcomes to the underlying EEG signal, Figure 7 shows the condition-averaged EEG power spectral density and Figure 8 the corresponding scalp topographic power maps for the reduced low-versus-high scenario (baseline, low, high).

### 3.3. The 6 s Baseline vs. 3 s Overlap Run

The overlap comparison is designed as a controlled segmentation ablation rather than as a separate analytical pipeline. All upstream quality-control rules, preprocessing choices, feature definitions, class scenarios, and model search settings were held constant across runs; only the temporal segmentation scheme differed. The comparison therefore isolates whether denser sampling of the same 6 s parent interval materially changes benchmark outcomes.

Because the overlap profile generates multiple sub-windows from the same parent trial, it increases temporal sampling density without creating the same amount of independent evidence as a true threefold increase in trials. Any performance gain should therefore be interpreted as a segmentation effect under denser temporal sampling, not as a simple consequence of additional independent observations.

Upstream inputs were matched across runs: both profiles processed the same 18 subjects and 1260 trial rows, and both retained the same modality-specific carry-forward participant sets. The overlap profile increased row density close to the expected threefold level at the epoch, feature-table, and machine learning-table stages, while changing retention proportions only modestly. Crucially, the downstream subject omissions were unchanged by overlap segmentation, indicating that the comparison reflects denser temporal sampling within the same effective participant set rather than a shift in cohort composition.

Across the matched scenario–dataset–protocol cells, mean balanced accuracy increased from 0.324 in the 6 s baseline run to 0.380 in the overlap run, and mean macro-F1 increased from 0.296 to 0.363. Forty-six of the 60 matched cells improved, indicating that the overlap representation was usually beneficial within the present benchmark despite the dependence between neighbouring windows.

**Figure 9:**
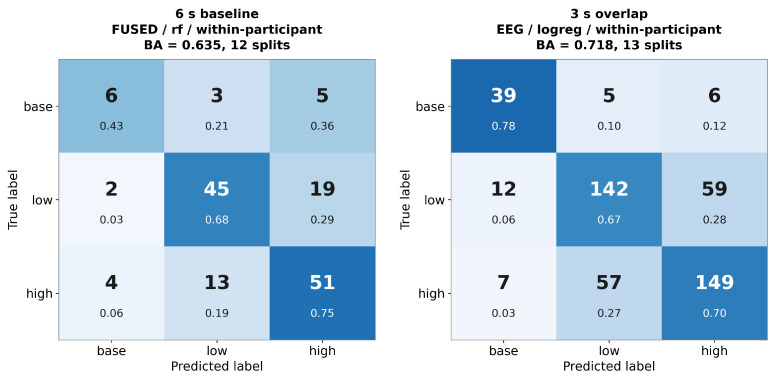
Confusion matrices for the best-performing 6 s baseline and 3 s overlap pipelines, both from the reduced three-class low-vs-high scenario omitting the hardest band. Class labels are abbreviated baseline/low/high; each cell gives the pooled count with the row-normalised recall beneath it, and shading encodes recall, so a dark leading diagonal indicates correct classification.

Across both profiles, the strongest baseline result reached a balanced accuracy of 0.635, while the strongest overlap result reached 0.718 (delta +0.083). Figure 9 compares the best overall 6 s baseline pipeline, fused random forest under within-participant validation, against the stronger overlap winner, EEG logistic regression under within-participant validation, both from the reduced three-class low-vs-high scenario omitting the hardest band.

Because these two headline winners use different dataset–model combinations, the figure should not be interpreted as isolating the segmentation effect by itself. The controlled segmentation evidence is instead the matched 60-cell comparison reported below, where class construction, dataset, validation protocol, feature definitions, and model search settings are held constant across the 6 s and 3 s profiles.

**Figure 10:**
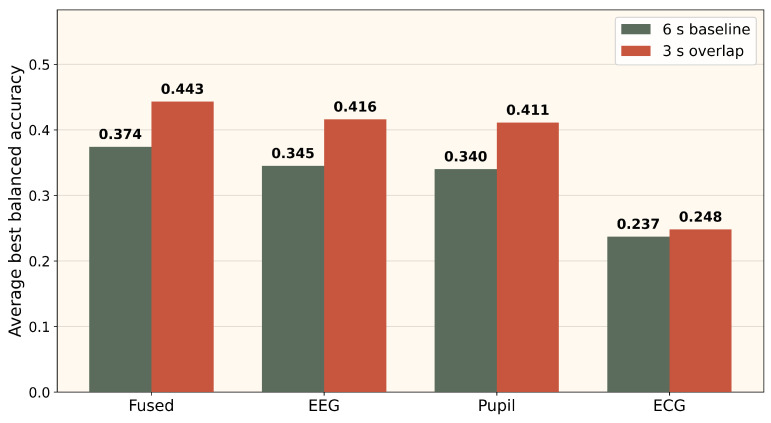
Average best-balanced accuracy for fused, EEG, pupil, and ECG datasets under the 6 s baseline and 3 s overlap runs, highlighting whether denser windowing changes the modality ranking.

The modality ranking stayed the same after overlap segmentation, with all four datasets improving in average best-balanced accuracy: fused 0.374 to 0.443 (+0.070), EEG 0.345 to 0.416 (+0.070), pupil 0.340 to 0.411 (+0.071), and ECG 0.237 to 0.248 (+0.012). The largest gains therefore came from fused, pupil, and EEG, while ECG changed only marginally. As shown in Figure 10, fused representations still led overall, followed by EEG, pupil, and ECG, although the magnitude of improvement differed by modality.

At the protocol level, overlap was not uniformly beneficial. Within-participant improved from 0.382 to 0.466 (+0.084), pooled-stratified improved from 0.321 to 0.409 (+0.087), and group-holdout declined slightly from 0.269 to 0.264 (−0.005). The current benchmark therefore suggests that overlap helps personalised and pooled decoding more than strict unseen-participant transfer.

**Figure 11:**
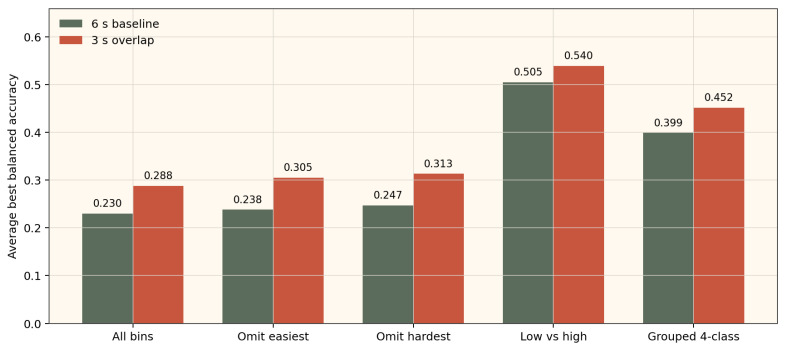
Average best-balanced accuracy for baseline all bins, baseline omit easiest, baseline omit hardest, baseline grouped 4class omit hardest, and baseline low high omit hardest in the 6 s baseline and overlap runs.

Scenario-level mean performance improved in all five label constructions: baseline all bins: 0.230 to 0.288 (+0.058); baseline omit easiest: 0.238 to 0.305 (+0.067); baseline omit hardest: 0.247 to 0.313 (+0.066); baseline grouped 4class omit hardest: 0.399 to 0.452 (+0.053); and baseline low high omit hardest: 0.505 to 0.540 (+0.035). The overlap run kept fused random forest as the winner for the all-bins, omit-easiest, and 4class scenarios, but winner identities changed in other settings: baseline omit hardest shifted from pupil random forest to EEG multilayer perceptron, and baseline low high omit hardest shifted from fused random forest to EEG logistic regression. Across the full matched grid, the winning model identity changed in 27 of 60 cells. Figure 11 shows that all five scenario constructions improved under overlap segmentation, although the size of the gain was larger for the higher-cardinality settings than for the reduced low-versus-high scenario.

Taken together, these results indicate that segmentation strategy is a meaningful benchmark variable rather than a minor preprocessing choice. In this dataset, denser overlapping windows improved most matched benchmark cells while preserving the same downstream subject set, but the gains were concentrated in within-participant and pooled-stratified evaluation rather than in strict unseen-participant transfer. The implication is not that overlap solves the harder problem of subject-independent generalisation, but that segmentation changes the temporal representation on which benchmark conclusions are based.

Table 6 summarises the like-for-like comparison across the 60 matched cells, including mean balanced accuracy and macro-F1 changes, while Table 7 summarises how scenario winners changed between the 6 s baseline and 3 s overlap runs.

#### Statistical Significance Procedures (Stage 7)

The inferential statistics summarised below are produced by a single dedicated stage (stage7_significance) of the released analysis pipeline, which loads the per-cell aggregate results emitted by Stage 6, derives outer-fold balanced-accuracy distributions and per-fold confusion matrices, and writes a machine-readable significance summary (significance_summary.json) alongside a markdown report. To make split stability auditable, the same JSON and markdown outputs include, for every scenario–dataset–protocol cell, the number of outer folds, mean balanced accuracy, fold-level SD and SE, *t*-based 95% confidence interval, and bootstrap 95% confidence interval. Stage 7 implements four procedures: a paired Wilcoxon signed-rank test of the per-cell best-model mean balanced accuracies for the 3 s vs. 6 s contrast, a 2000-sample nonparametric bootstrap of the per-cell mean balanced accuracy over outer-fold scores (percentile-method 95% CIs), a one-sided Wilcoxon signed-rank test of outer-fold balanced accuracies against the per-scenario chance level $1/n_{\mathrm{classes}}$, and a 10,000-iteration label-shuffle permutation test on the held-out predicted-label vectors reconstructed from the per-fold confusion matrices, with Bonferroni correction over the full 120-cell grid. The same stage also emits the paired Wilcoxon test on baseline-vs-task heart rate referenced in Section 2.2 and Figure 2. Concretely, the 3 s overlap improvement was significantly larger than the 6 s baseline (W=1636, $p=5.55\times 10^{-8}$, paired n=60). The one-sided Wilcoxon vs chance passed at p<0.05 for 111 of 120 best-model cells (92.5%; uncorrected). The label-shuffle permutation test returned $p_{\mathrm{perm}}<10^{-4}$ for all five 6 s scenario winners and all five 3 s overlap scenario winners, which survived Bonferroni correction over the 120 cells (threshold of $4.17\times 10^{-4}$). Across the broader 120-cell set, 103 cells survived Bonferroni correction and 100 reached the plus-one empirical floor of the 10,000-permutation null (*p* ≤ 1/10,001), indicating that the headline scenario-winner findings are robust to multiple-comparison correction even when per-cell fold counts are modest.

## 4. Discussion

### 4.1. Validation Protocol Effects

These benchmark results reinforce that validation protocol is not a reporting detail but a major determinant of the apparent difficulty of workload decoding. Within-participant evaluation produced the highest scores across all scenario–dataset combinations, pooled-stratified evaluation remained intermediate, and group-holdout provided the most conservative estimate of transfer to unseen participants. The size of that gap suggests that a substantial part of the signal captured by the current feature set is participant-specific, which is why results from less restrictive splits should not be read as evidence of robust subject-independent generalisation [3,11,25,28].

### 4.2. Modality and Fusion Patterns

The modality pattern is also informative. Fused representations were strongest overall and dominated most scenario–protocol cells, indicating that complementary information is being captured across central and peripheral measures rather than by a single sensing stream alone. Among the unimodal inputs, EEG was consistently strongest, pupil remained competitive enough to win one scenario, and ECG lagged behind as a standalone source. That ordering is consistent with the multimodal workload literature, where cortical measures often carry the clearest discriminative structure while autonomic and ocular signals add useful but incomplete information [3,22,23,24].

### 4.3. Model Family Comparisons

Model family comparisons point in the same direction. Classical models outperformed the tested neural architectures across most benchmark cells, and random forest alone accounted for half of all winning model slots. In a dataset of this size with heavily engineered summary features, that is not an especially surprising result: the limiting factor appears to be representation design and evaluation protocol more than raw model capacity. Neural wins in ECG and pooled-stratified settings remain worth reporting, but they are narrower exceptions than a basis for a general claim that deeper architectures are better suited to this benchmark [3,28].

### 4.4. Comparison with Prior Workload Benchmarks

Direct comparison with prior workload-classification studies is inherently approximate, because reported metrics, class constructions, validation regimes, and tasks differ across the literature, which is precisely the methodological problem the present benchmark sets out to make explicit [1,2,3]. Three reference points nonetheless help anchor the present results. First, the Pušica et al. 2025 systematic review of EEG-based mental workload classification highlights that reported accuracies range very widely depending on task structure, from ∼70% on a 36-class intelligence test paradigm up to ∼90% in a binary single-task setting, and explicitly notes that “no standardized benchmark task exists”, so per-paper accuracy figures are not directly transferable [1]. Second, in the most direct precursor for the present arithmetic paradigm, Spüler et al. (2016) and Walter et al. (2017) reported cross-subject workload regression performance with average correlations around r≈0.82 between predicted and observed workload, which is consistent in spirit with the present finding that EEG carries substantial subject-shared workload information even under conservative cross-validation, although the present work casts the problem as classification rather than regression and adds ECG and pupillometry [8,9]. Third, in the multimodal six-task, six-sensor study of Mark et al. (2024) [22], EEG and fNIRS jointly explained the largest share of workload variance (47.5% and 48.0% respectively), with cardiac and ocular measures contributing markedly less. The modality ordering reported here—fused > EEG > pupil > ECG—is consistent with the prior pattern under the constraints of the present arithmetic dataset and feature set [21,22,23]. The comparative absolute scores reported here, including a top within-participant balanced accuracy of 0.718 for the reduced three-class scenario under 3 s overlap segmentation, sit within the band of plausible outcomes for graded multimodal workload classification given small participant pools and conservative participant-aware evaluation, while remaining below the headline numbers reported in less restrictive single-task or binary benchmarks [1,28].

### 4.5. Segmentation Effects

The overlap ablation sharpens the main methodological message. Denser 3 s windows improved most matched cells and raised average scores for fused, EEG, and pupil inputs, yet those gains were concentrated in within-participant and pooled-stratified evaluation while group-holdout remained flat to slightly worse. That pattern suggests overlap mainly strengthens local temporal continuity and participant-specific regularities rather than solving the harder problem of cross-subject transfer. For that reason, the overlap profile is best treated as a useful segmentation benchmark and sensitivity analysis, not as standalone evidence that denser windowing improves subject-independent workload decoding.

### 4.6. Limitations

Several limitations should be made explicit. First, the cohort is small (18 participants in pre-screening; 13–15 retained per modality after participant-level QC). Within-participant evaluation in particular relies on ∼65–70 windows per subject in the 6 s profile against feature dimensionalities up to 153 (EEG) and 196 (fused), so within-participant scores should be read as personalised upper-bound estimates with non-trivial overfitting risk rather than as evidence of a general decoding capability. Second, *Q*-value is an objective descriptor of nominal arithmetic difficulty, not a direct measurement of each participant’s experienced cognitive load. Individual arithmetic skill, familiarity, fatigue, and strategy may alter the workload induced by the same nominal item, so the models may partly classify structured task difficulty and participant-specific response patterns rather than cognitive load alone. This caution is also consistent with recent pupillometry work showing that task-load effects can vary across task modality and physiological measure, motivating multivariate workload assessment rather than direct equivalence between nominal task difficulty and experienced workload [29]. Third, while the headline scenario winners now carry bootstrap 95% confidence intervals and a paired Wilcoxon signed-rank test supports the 3 s vs 6 s contrast at $p<10^{-7}$, full per-cell multiple-comparison correction across the 60 scenario–dataset–protocol cells has not yet been applied. The per-cell significance-against-chance results (111 of 120 best-model cells at p<0.05, uncorrected) should therefore be read as supportive rather than definitive; under Bonferroni correction over the 120 tests ($p<4.17\times 10^{-4}$), the 10 scenario-winner cells survive on the basis of the 10,000-permutation label-shuffle test ($p_{\mathrm{perm}}<10^{-4}$ for every winner under both 6 s and 3 s overlap segmentations), and a smaller subset of the remaining cells survive the same correction; surviving cells are concentrated in the within-participant protocol and the lower-cardinality scenarios. Fourth, the pooled-stratified protocol allows the same participant to contribute to both training and test partitions and is included as an explicit demonstration of how permissive splitting inflates apparent performance, not as a recommended evaluation regime for deployment claims; readers should treat group-holdout as the only protocol that quantifies transfer to unseen individuals. Fifth, the deep learning hyperparameter sweep (≤6 random combinations × three-fold inner CV per family) is shallower than the classical-model sweep, so the classical-versus-neural comparison is conservative against neural architectures rather than balanced; deeper neural sweeps remain a useful follow-up. Sixth, the paradigm is mental arithmetic on a single open dataset; generalisation of the modality, segmentation, and validation conclusions to other workload paradigms (e.g., MATB, n-back, naturalistic tasks) and to other recording rigs requires explicit external validation. Finally, ICA was deliberately omitted in the reported runs to minimise pipeline branching; an ICA-on/ICA-off ablation is left as an explicit sensitivity analysis. This omission also means that residual ocular artefacts, especially blinks and frontal eye-movement activity, may remain in frontal EEG features; this is particularly relevant because pupillometry and gaze-derived measures are analysed in parallel. The benchmark infrastructure released alongside this paper is designed to make all of these follow-up analyses straightforward to add.

## 5. Conclusions

This study presented a reproducible benchmark for graded physiological workload classification on the open ds007262 dataset [7]. The conclusions below should be read as conclusions for this dataset, feature set, and analysis procedure rather than universal rankings of workload modalities or classifier families. Across the benchmark suite, fused representations generally performed best, EEG was the strongest unimodal modality, and classical machine learning models outperformed the tested deep learning approaches in most feature-based settings. Evaluation protocol materially changed interpretation: within-participant decoding produced the highest scores, pooled-stratified testing remained more optimistic than participant-aware splits, and group-holdout evaluation provided the clearest estimate of transfer to unseen individuals [3,11,25]. The controlled comparison between fixed 6 s trial windows and denser overlapping 3 s sub-windows further showed that segmentation is not a minor preprocessing choice: overlap improved most matched cells and raised mean balanced accuracy from 0.324 to 0.380, but those gains were concentrated in within-participant and pooled settings rather than strict unseen-participant transfer [20]. The work therefore contributes a transparent benchmark framework rather than a single best-model claim, providing a reproducible basis for future comparison of modality choice, fusion strategy, segmentation design, and validation regime on an open graded-workload dataset, and extending arithmetic-based workload modelling toward a clearer benchmark setting for adaptive educational and neuroergonomic applications [7,8,9]. The present study does not test a workplace redesign, tool arrangement, safety outcome, comfort rating, efficiency intervention, or well-being endpoint directly. Its ergonomic significance is therefore methodological: workload-aware ergonomic systems require classifiers whose reported performance can be interpreted under the deployment question being asked. A model that works only within participants may be useful for personalised adaptive interfaces or training feedback, whereas a model claimed for new workers, workstations, or work-system designs requires participant-aware validation. By separating modality, fusion, segmentation, and validation effects, the framework helps identify which physiological inputs and evaluation protocols are credible candidates for later ergonomic studies of workplace tools, interfaces, and work systems, while leaving direct safety, comfort, efficiency, and well-being validation to future task-specific deployments; recent human factors and industrial studies similarly link physiological workload assessment to surgical training, instruction design, operational efficiency, and worker-centred system design [6,30].

## Figures and Tables

**Table 1:** Pipeline execution summary; each row corresponds to one stage*N*_*.py module in the released codebase [15].

Stage	Purpose	Key Outputs
0	Build canonical trial table from BIDS events and trial timings	Trial-table TSV; trial-table summary JSON
1	Participant-level QC and modality-specific carry-forward decisions	QC subject table; QC summary JSON; QC figures
2	Preprocess EEG, ECG, and pupil streams; produce cleaned derivatives	Cleaned derivatives; preprocess log and summary JSON
3	Segment trial-aligned analysis windows; reject failed epochs	Epoch NPZ files; epoch manifest; drop log; segmentation summary
4	Extract unimodal EEG, ECG, and pupil features per retained window	features_eeg.tsv; features_ecg.tsv; features_pupil.tsv; feature summary
5	Build unimodal and early-fusion ML tables; emit split manifests	Unimodal and fused TSVs; split manifest JSON; fusion summary
6	Train and evaluate classical and neural models; build report assets	ML result JSON; summary markdown; confusion PNGs
7	Pair Wilcoxon, bootstrap CIs, label-shuffle permutation, and Bonferroni correction over the matched cell grid	Significance summary JSON and markdown

**Table 2:** Per-participant raw acquisition counts for the EEG/ECG mobile recording device, with the Stage 1 union-gate QC outcome (‘retained’ = carried forward into at least one modality set; ‘rejected’ = removed from all modelling).

Participant	Recorded ^1^	Dropped ^2^	Drop Rate (%)	Stage 1 QC ^3^
sub-001	172,704	236	0.14	retained
sub-003	189,126	723	0.38	retained
sub-004	193,128	1152	0.59	retained
sub-005	230,124	1204	0.52	retained
sub-006 ^4^	206,130	653	0.32	rejected
sub-007	214,158	542	0.25	retained
sub-008 ^4^	384,246	2897	0.75	rejected
sub-009	214,458	609	0.28	retained
sub-010	267,384	701	0.26	retained
sub-011	299,502	1490	0.50	retained
sub-012	198,000	1226	0.62	retained
sub-013	200,502	1478	0.73	retained
sub-014	223,128	628	0.28	retained
sub-015	205,530	913	0.44	retained
sub-016	284,628	1087	0.38	retained
sub-018	227,748	560	0.25	retained
sub-019	208,632	1035	0.49	retained
sub-020	205,968	1409	0.68	retained
**Total (*n* = 18)**	**4,125,096**	**18,543**	**0.45**	**16 retained, 2 rejected**

^1^ Number of samples written to the BIDS continuous channels during the arithmetic protocol; both EEG and ECG share the same 250 Hz mobile acquisition device, so the counts apply jointly to both modalities. ^2^ Samples flagged as acquisition gaps by the Lab Streaming Layer converter (typically brief Wi-Fi/UDP or buffer hiccups) excluded from epoching. ^3^ Outcome of the Stage 1 participant-level QC gate: participants marked ‘retained’ are carried forward into at least one modality-specific set, whereas ‘rejected’ participants are removed from all modelling. Some union-retained participants are nonetheless excluded from individual modalities by the later modality-specific screening (see the Data Retention text). ^4^ Rejected by Stage 1 QC: sub-006 for an anomalous started_arithmetic marker count; sub-008 for high mean and p95 dropped-sample rates combined with low pupil mean confidence (0.471). Values in the final row (**Total**) are set in bold to denote the cohort totals and overall QC outcome.

**Table 3:** Class-construction scenarios and their mappings from the seven arithmetic difficulty bands ($Q_{1}$–$Q_{7}$, ordered easiest to hardest) plus baseline fixations onto class labels. The numeric *Q*-value ranges are $Q_{1}=0.6$–1.5, $Q_{2}=1.5$–2.4, $Q_{3}=2.4$–3.3, $Q_{4}=3.3$–4.2, $Q_{5}=4.2$–5.1, $Q_{6}=5.1$–6.0, and $Q_{7}=6.0$–6.9. Class counts exclude any band that is omitted from the scenario.

Scenario	Classes	Mapping
all_bins	8	baseline; $Q_{1}$; $Q_{2}$; $Q_{3}$; $Q_{4}$; $Q_{5}$; $Q_{6}$; $Q_{7}$
omit_easiest	7	baseline; $Q_{2}$; $Q_{3}$; $Q_{4}$; $Q_{5}$; $Q_{6}$; $Q_{7}$ ($Q_{1}$ dropped)
omit_hardest	7	baseline; $Q_{1}$; $Q_{2}$; $Q_{3}$; $Q_{4}$; $Q_{5}$; $Q_{6}$ ($Q_{7}$ dropped)
4class_omit_hardest	4	baseline; low ($Q_{1}$–$Q_{2}$); medium ($Q_{3}$–$Q_{4}$); high ($Q_{5}$–$Q_{6}$); $Q_{7}$ dropped
low_high_omit_hardest	3	baseline; low ($Q_{1}$–$Q_{3}$); high ($Q_{4}$–$Q_{6}$); $Q_{7}$ dropped

**Table 4:** Best-balanced accuracy by modality and validation protocol (6 s baseline, averaged across five class scenarios). Cells show mean BA (SD; SE; 95% CI) across class scenarios.

Modality	Within-Part.	Pooled-Strat.	Group-Holdout	Mean BA
Fused	0.446 (0.130; 0.058; [0.284, 0.607])	0.366 (0.139; 0.062; [0.194, 0.538])	0.309 (0.127; 0.057; [0.151, 0.466])	0.374
EEG	0.406 (0.150; 0.067; [0.219, 0.592])	0.354 (0.154; 0.069; [0.162, 0.545])	0.277 (0.127; 0.057; [0.120, 0.434])	0.345
Pupil	0.411 (0.106; 0.047; [0.279, 0.542])	0.343 (0.129; 0.057; [0.183, 0.503])	0.267 (0.114; 0.051; [0.126, 0.409])	0.340
ECG	0.266 (0.116; 0.052; [0.121, 0.411])	0.222 (0.096; 0.043; [0.102, 0.341])	0.222 (0.095; 0.043; [0.104, 0.341])	0.237

**Table 5:** Classical vs. neural model family comparison across all 60 best-model cells (6 s baseline run).

Metric	Classical Models	Neural Models
Best-model cell wins (of 60)	47	13
Mean family-best BA	0.322	0.295
Wins on fused data	14	1
Wins on EEG data	12	3
Wins on pupil data	15	0
Wins on ECG data	6	9
Wins: within-participant	18	2
Wins: pooled-stratified	12	8
Wins: group-holdout	17	3
Top classical model (wins)	Random Forest (30)	–
Top neural model (wins)	–	Multilayer perceptron (7)

**Table 6:** Like-for-like run comparison. The Wilcoxon row is a paired signed-rank test across the 60 matched scenario–dataset–protocol cells, comparing the best-model mean balanced accuracy under 3 s overlap versus the 6 s baseline.

Metric	Value
Matched comparison cells	60
Mean balanced accuracy: 6 s baseline	0.324
Mean balanced accuracy: 3 s overlap	0.380
Mean balanced accuracy delta	+0.056
Mean macro-F1: 6 s baseline	0.296
Mean macro-F1: 3 s overlap	0.363
Mean macro-F1 delta	+0.068
Matched cells improved	46
Matched cells worsened	14
Matched cells unchanged	0
Winning model changed	27
Wilcoxon paired (3 s > 6 s)	W=1636, $p=5.55\times 10^{-8}$

**Table 7:** Scenario winners with bootstrap 95% confidence intervals on the mean balanced accuracy across outer-fold splits (2000-sample nonparametric bootstrap; $n_{\mathrm{splits}}$ depends on protocol: within-participant ∼13–15, pooled-stratified 5, group-holdout 15). All scenario winners are significantly above chance at p<0.001 (one-sided Wilcoxon signed-rank against the chance level of $1/n_{\mathrm{classes}}$).

Scenario	6 s Winner	6 s BA [95% CI]	3 s Overlap Winner	3 s BA [95% CI]	Delta
all_bins	fused/within/rf	0.344 [0.281, 0.417]	fused/within/rf	0.451 [0.403, 0.496]	+0.107
omit_easiest	fused/within/rf	0.393 [0.327, 0.458]	fused/within/rf	0.474 [0.403, 0.547]	+0.081
omit_hardest	pupil/within/rf	0.362 [0.305, 0.414]	eeg/within/mlp	0.464 [0.431, 0.494]	+0.102
4class	fused/within/rf	0.524 [0.444, 0.611]	fused/within/rf	0.640 [0.579, 0.700]	+0.116
low_high	fused/within/rf	0.635 [0.556, 0.722]	eeg/within/logreg	0.718 [0.656, 0.777]	+0.083

## Data Availability

The original physiological recordings analysed in this study are openly available in OpenNeuro at https://doi.org/10.18112/openneuro.ds007262.v1.1.0 (accession ds007262, version 1.1.0; accessed on 2 July 2026) [7]. The complete analysis pipeline is openly available on GitHub (https://github.com/LMBooth/Arithmetic_Workload_Estimation, accessed on 2 July 2026) and archived on Zenodo at version 0.0.4, DOI https://doi.org/10.5281/zenodo.20162864 (accessed on 8 June 2026) [15], released under the CC0 1.0 Universal licence. The two checked-in YAML configuration profiles reproduce the Stage 0 through Stage 6 results from a single command, and Stage 7 (stage7_significance) reproduces every inferential statistic reported in Section 3.3, including the paired Wilcoxon W=1636, the bootstrap CIs in Table 7, and the label-shuffle permutation *p*-values.

## Appendix A. Feature Definitions

This appendix lists the full set of EEG, ECG, and pupillometry features computed for each retained window and used for downstream modelling.

Table A1 summarises how the reported feature dimensionalities are obtained from the implemented feature groups. The fused modelling table contains the concatenated EEG, ECG, and pupillometry feature columns after non-feature metadata columns are removed.

**Table A1:** Feature count derivation for the machine learning tables.

Modality	Count Derivation	Total
EEG	Five fused compatible ROIs with 27 ROI-level summaries each (5×27=135), plus 15 ROI-by-band baseline-delta features and three frontal/Fz channel-specific features.	153
ECG	Sixteen HR/HRV/quality features plus two baseline-referenced delta features.	18
Pupillometry	Twenty-three pupil, gaze, confidence, velocity, and coverage features plus two baseline-referenced delta features.	25
Fused	Concatenation of the 153 EEG, 18 ECG, and 25 pupillometry feature columns after metadata exclusion (153+18+25=196).	196

**Table A2:** EEG feature descriptions and equations.

Description	Equation
Absolute power in a frequency band for a region/ROI	$\sum_{f\in \mathrm{band}}{\mathrm{PSD}}_{\mathrm{ROI}}\left(f\right)$
Change from baseline in absolute band power (task minus baseline)	$P_{\mathrm{abs}}^{\mathrm{task}}\left(\mathrm{band},\mathrm{ROI}\right)-P_{\mathrm{abs}}^{\mathrm{base}}\left(\mathrm{band},\mathrm{ROI}\right)$
Band power normalised by total band power in that ROI	$P_{\mathrm{abs}}\left(\mathrm{band},\mathrm{ROI}\right)/\sum_{k\in B}P_{\mathrm{abs}}\left(k,\mathrm{ROI}\right)$
Ratio of absolute band power between two bands	$P_{\mathrm{abs}}\left({\mathrm{band}}_{1},\mathrm{ROI}\right)/P_{\mathrm{abs}}\left({\mathrm{band}}_{2},\mathrm{ROI}\right)$
Signal variance (activity)	var(x)
Mobility (Hjorth)	$\sqrt{\mathrm{var}\left(\dot{x}\right)/\mathrm{var}\left(x\right)}$
Complexity (Hjorth)	$\sqrt{\mathrm{var}\left(\ddot{x}\right)/\mathrm{var}\left(\dot{x}\right)}/\mathrm{Mobility}\left(\mathrm{ROI}\right)$
Sum of absolute sample-to-sample changes	$\sum_{t=2}^{T}\left|x_{t}-x_{t-1}\right|$
Mean absolute amplitude	$\frac{1}{T}\sum_{t=1}^{T}\left|x_{t}\right|$
Mean of squared amplitude	$\frac{1}{T}\sum_{t=1}^{T}x_{t}^{2}$
Median of squared amplitude	$\mathrm{median}{\left\{x_{t}^{2}\right\}}_{t=1}^{T}$
Root mean square amplitude	$\sqrt{\frac{1}{T}\sum_{t=1}^{T}x_{t}^{2}}$
Spectral entropy (normalised in band)	$-\sum_{i}p_{i}\ln p_{i}$
Slope sign changes (count)	$\sum_{t=2}^{T-1}I\left[\left(x_{t}-x_{t-1}\right)\left(x_{t}-x_{t+1}\right)>0\right]$
SSC per second	$\mathrm{SSC}\left(\mathrm{ROI}\right)/{\mathrm{duration}}_{s}$
Variance in amplitude	$E[{\left(x-E\left[x\right]\right)}^{2}]$
Sign changes (count)	$\sum_{t=2}^{T}I\left[\mathrm{sign}\left(x_{t}\right)\neq \mathrm{sign}\left(x_{t-1}\right)\right]$
ZC per second	$\mathrm{ZC}\left(\mathrm{ROI}\right)/{\mathrm{duration}}_{s}$

Repeated features (per ROI, per band) include absolute band power, baseline-delta absolute band power, relative band power, and band power ratios. Channel-specific features that are not ROI-aggregated are frontal alpha asymmetry (F4 vs. F3) and Fz theta absolute and relative power.

**Table A3:** ECG feature descriptions and equations.

Description	Equation
Number of detected ECG peaks in the segment	$N_{\mathrm{peaks}}=\mathrm{count}\left(\mathrm{peaks}\right)$
Maximum heart rate (bpm)	${\mathrm{HR}}_{\max}=\max\left({\mathrm{HR}}_{i}\right)$
Mean heart rate (bpm)	${\mathrm{HR}}_{\mathrm{mean}}=\mathrm{mean}\left({\mathrm{HR}}_{i}\right)$
Δ mean heart rate (task − baseline)	$\Delta {\mathrm{HR}}_{\mathrm{mean}}={\mathrm{HR}}_{\mathrm{mean},\mathrm{task}}-{\mathrm{HR}}_{\mathrm{mean},\mathrm{base}}$
Median heart rate (bpm)	${\mathrm{HR}}_{\mathrm{median}}=\mathrm{median}\left({\mathrm{HR}}_{i}\right)$
Minimum heart rate (bpm)	${\mathrm{HR}}_{\min}=\min\left({\mathrm{HR}}_{i}\right)$
Standard deviation of heart rate (bpm)	${\mathrm{HR}}_{\mathrm{std}}=\mathrm{std}\left({\mathrm{HR}}_{i}\right)$
NN50	$\mathrm{NN}50=\mathrm{count}(|{\mathrm{RR}}_{i}-{\mathrm{RR}}_{i-1}|>50\;\mathrm{ms})$
pNN50 (%)	$\mathrm{pNN}50=100\cdot \mathrm{NN}50/(N_{\mathrm{RR}}-1)$
ECG quality flag (categorical)	n/a
RMSSD (ms)	$\mathrm{RMSSD}=\sqrt{\mathrm{mean}({\left({\mathrm{RR}}_{i}-{\mathrm{RR}}_{i-1}\right)}^{2})}$
Δ RMSSD (ms)	$\Delta \mathrm{RMSSD}={\mathrm{RMSSD}}_{\mathrm{task}}-{\mathrm{RMSSD}}_{\mathrm{base}}$
RR coverage	${\mathrm{RR}}_{\mathrm{cov}}=N_{\mathrm{valid}\;\mathrm{RR}}/N_{\mathrm{expected}\;\mathrm{RR}}$
RR coefficient of variation	${\mathrm{RR}}_{\mathrm{CV}}=\mathrm{std}\left(\mathrm{RR}\right)/\mathrm{mean}\left(\mathrm{RR}\right)$
RR interquartile range (ms)	${\mathrm{RR}}_{\mathrm{IQR}}=P_{75}\left(\mathrm{RR}\right)-P_{25}\left(\mathrm{RR}\right)$
Mean RR interval (ms)	${\mathrm{RR}}_{\mathrm{mean}}=\mathrm{mean}\left({\mathrm{RR}}_{i}\right)$
SDNN (ms)	$\mathrm{SDNN}=\mathrm{std}({\mathrm{RR}}_{i})$
Count of valid RR intervals	$N_{\mathrm{valid}\;\mathrm{RR}}$

**Table A4:** Pupillometry feature descriptions and equations.

Description	Equation
Total gaze path distance	$\mathrm{path}=\sum_{t}\sqrt{{\left(\Delta x_{t}\right)}^{2}+{\left(\Delta y_{t}\right)}^{2}}$
Mean horizontal gaze position	${\mathrm{mean}}_{x}=\mathrm{mean}\left(x_{t}\right)$
Std. horizontal gaze position	${\mathrm{std}}_{x}=\mathrm{std}\left(x_{t}\right)$
Mean vertical gaze position	${\mathrm{mean}}_{y}=\mathrm{mean}\left(y_{t}\right)$
Std. vertical gaze position	${\mathrm{std}}_{y}=\mathrm{std}\left(y_{t}\right)$
Area under pupil curve above mean	$\mathrm{AUC}=\int \max(\mathrm{pupil}\left(t\right)-\bar{\mathrm{pupil}},0)\;dt$
Mean pupil sample confidence	${\mathrm{conf}}_{\mathrm{mean}}=\mathrm{mean}\left({\mathrm{conf}}_{t}\right)$
Pupil interquartile range	$\mathrm{IQR}=P_{75}\left(\mathrm{pupil}\right)-P_{25}\left(\mathrm{pupil}\right)$
Low-confidence sample fraction	$\mathrm{low}\_\mathrm{conf}=\mathrm{count}({\mathrm{conf}}_{t}<\tau )/N$
Maximum pupil size	${\mathrm{pupil}}_{\max}$
Mean pupil size	${\mathrm{pupil}}_{\mathrm{mean}}$
Δ mean pupil size	$\Delta {\mathrm{pupil}}_{\mathrm{mean}}={\mathrm{pupil}}_{\mathrm{mean},\mathrm{task}}-{\mathrm{pupil}}_{\mathrm{mean},\mathrm{base}}$
Median pupil size	${\mathrm{pupil}}_{\mathrm{median}}$
Minimum pupil size	${\mathrm{pupil}}_{\min}$
10th percentile pupil size	${\mathrm{pupil}}_{p10}$
90th percentile pupil size	${\mathrm{pupil}}_{p90}$
Peak pupil dilation amplitude	$\mathrm{peak}\_\mathrm{dil}=\max({\mathrm{pupil}}_{t})-\mathrm{baseline}$
Δ peak pupil dilation	Δpeak_dil
Time to peak pupil response (s)	$t_{\mathrm{peak}}=\arg\max_{t}{\mathrm{pupil}}_{t}$
Linear pupil slope (per s)	slope=d(pupil)/dt(linearfit)
Std. pupil size	${\mathrm{pupil}}_{\mathrm{std}}$
Task-evoked pupillary response (TEPR) latency (s)	${\mathrm{TEPR}}_{\mathrm{lat}}=t_{\mathrm{peak}}-t_{\mathrm{onset}}$
Coverage	$N_{\mathrm{valid}}/N_{\mathrm{expected}}$
Maximum absolute pupil velocity	${\mathrm{vel}}_{\max}=\max\left|d\mathrm{pupil}/dt\right|$
Mean absolute pupil velocity	${\mathrm{vel}}_{\mathrm{mean}}=\mathrm{mean}\left|d\mathrm{pupil}/dt\right|$

In Table A4, $x_{t}$ and $y_{t}$ denote the cleaned normalised horizontal and vertical gaze coordinates at sample *t*, ${\mathrm{pupil}}_{t}$ denotes cleaned pupil size, ${\mathrm{conf}}_{t}$ denotes the Pupil Labs confidence value, and $t_{\mathrm{onset}}$ denotes the start of the analysed arithmetic window.

## Appendix B. Full Per-Cell Best-Pipeline Table

**Table A5:** Best pipeline per scenario–dataset–protocol combination (6 s baseline run).

Scenario	Dataset	Protocol	Best Model	Bal. Acc.	Macro F1
all_bins	EEG	within_participant	rf	0.303	0.254
all_bins	EEG	pooled_stratified	rf	0.237	0.227
all_bins	EEG	group_holdout	rf	0.170	0.127
all_bins	ECG	within_participant	svm	0.183	0.140
all_bins	ECG	pooled_stratified	transformer	0.144	0.109
all_bins	ECG	group_holdout	knn	0.142	0.126
all_bins	Pupil	within_participant	rf	0.321	0.277
all_bins	Pupil	pooled_stratified	rf	0.246	0.241
all_bins	Pupil	group_holdout	rf	0.183	0.157
all_bins	Fused	within_participant	rf	0.344	0.320
all_bins	Fused	pooled_stratified	rf	0.275	0.267
all_bins	Fused	group_holdout	rf	0.216	0.169
omit_easiest	EEG	within_participant	svm	0.297	0.246
omit_easiest	EEG	pooled_stratified	mlp	0.248	0.252
omit_easiest	EEG	group_holdout	logreg	0.201	0.160
omit_easiest	ECG	within_participant	transformer	0.187	0.126
omit_easiest	ECG	pooled_stratified	mlp	0.164	0.160
omit_easiest	ECG	group_holdout	mlp	0.167	0.145
omit_easiest	Pupil	within_participant	rf	0.333	0.298
omit_easiest	Pupil	pooled_stratified	rf	0.229	0.224
omit_easiest	Pupil	group_holdout	rf	0.177	0.152
omit_easiest	Fused	within_participant	rf	0.393	0.346
omit_easiest	Fused	pooled_stratified	rf	0.249	0.244
omit_easiest	Fused	group_holdout	mlp	0.214	0.179
omit_hardest	EEG	within_participant	gaussian_nb	0.297	0.248
omit_hardest	EEG	pooled_stratified	mlp	0.261	0.262
omit_hardest	EEG	group_holdout	rf	0.201	0.153
omit_hardest	ECG	within_participant	logreg	0.187	0.157
omit_hardest	ECG	pooled_stratified	mlp	0.159	0.153
omit_hardest	ECG	group_holdout	logreg	0.165	0.142
omit_hardest	Pupil	within_participant	rf	0.362	0.304
omit_hardest	Pupil	pooled_stratified	rf	0.281	0.276
omit_hardest	Pupil	group_holdout	rf	0.206	0.176
omit_hardest	Fused	within_participant	rf	0.333	0.309
omit_hardest	Fused	pooled_stratified	rf	0.284	0.277
omit_hardest	Fused	group_holdout	rf	0.236	0.192
4class	EEG	within_participant	rf	0.518	0.476
4class	EEG	pooled_stratified	mlp	0.436	0.439
4class	EEG	group_holdout	svm	0.343	0.291
4class	ECG	within_participant	transformer	0.335	0.267
4class	ECG	pooled_stratified	transformer	0.277	0.214
4class	ECG	group_holdout	knn	0.272	0.250
4class	Pupil	within_participant	svm	0.469	0.422
4class	Pupil	pooled_stratified	rf	0.443	0.436
4class	Pupil	group_holdout	rf	0.337	0.307
4class	Fused	within_participant	rf	0.524	0.478
4class	Fused	pooled_stratified	svm	0.458	0.458
4class	Fused	group_holdout	rf	0.378	0.346
low_high	EEG	within_participant	logreg	0.615	0.577
low_high	EEG	pooled_stratified	svm	0.588	0.570
low_high	EEG	group_holdout	svm	0.469	0.410
low_high	ECG	within_participant	rf	0.439	0.415
low_high	ECG	pooled_stratified	cnn1d	0.366	0.363
low_high	ECG	group_holdout	gru1d	0.367	0.313
low_high	Pupil	within_participant	decision_tree	0.568	0.518
low_high	Pupil	pooled_stratified	svm	0.516	0.514
low_high	Pupil	group_holdout	rf	0.434	0.407
low_high	Fused	within_participant	rf	0.635	0.601
low_high	Fused	pooled_stratified	rf	0.565	0.582
low_high	Fused	group_holdout	svm	0.500	0.486

## Appendix C. Per-Scenario Confusion Matrices

The main text reports confusion matrices for the headline three-class low_high_omit_hardest scenario (Figure 9), which is the clearest performance endpoint. For completeness, Figure A1 additionally shows the confusion matrix of the highest balanced-accuracy pipeline for each of the five class-construction scenarios defined in Table 3, all taken from the 6 s baseline run. The selected pipelines coincide with the best within-participant rows of Table A5: fused random forest for all_bins (balanced accuracy 0.344), omit_easiest (0.393), 4class_omit_hardest (0.524) and low_high_omit_hardest (0.635), and pupil random forest for omit_hardest (0.362). Cell counts are pooled across the evaluated splits and shading encodes row-normalised recall, so a clean leading diagonal indicates correct classification. The diagonal becomes progressively cleaner as class cardinality is reduced from eight to three, and the residual confusions remain concentrated between physiologically adjacent difficulty bands rather than being uniformly distributed, consistent with the graded nature of the difficulty labels.
